# HIV Related High Risk Behaviors and Willingness to Participate in HIV Vaccine Trials among China MSM by Computer Assisted Self-Interviewing Survey

**DOI:** 10.1155/2013/493128

**Published:** 2013-11-25

**Authors:** Zhenxing Chu, Junjie Xu, Kathleen Heather Reilly, Chunming Lu, Qinghai Hu, Ning Ma, Min Zhang, Jing Zhang, Yongjun Jiang, Wenqing Geng, Hong Shang

**Affiliations:** ^1^Key Laboratory of AIDS Immunology of Ministry of Health, Department of Laboratory Medicine, The First Hospital of China Medical University, Shenyang 110001, China; ^2^Collaborative Innovation Center for Diagnosis and Treatment of Infectious Diseases, Hangzhou 310000, China; ^3^National Center for AIDS/STD Control and Prevention, Chinese Center for Disease Control and Prevention, Beijing 102206, China; ^4^Liaoning Provincial CDC, Shenyang 110005, China

## Abstract

*Background*. The number of new HIV infections among MSM of China is rapidly increasing in recent years and behavioral interventions have had limited effectiveness. To control the HIV pandemic may lie in an HIV vaccine. This study examined the factors associated with willingness to participate (WTP) in HIV vaccine clinical trials among China MSM. *Methods*. A cross-sectional survey was carried out among MSM from three cities in northeast China. Questionnaires pertaining to MSM risk behavior and WTP in HIV vaccine trials were administered through computer assisted self-interviewing (CASI). *Results*. A total of 626 MSM participated in this survey. 54.8% had occasional male partners and 52.2% always used condoms with male sex partners. HIV prevalence was 5.0%. 76.7% were WTP in a preventive HIV vaccine clinical trial. Results showed that HIV vaccination is a means of protection for spouses and family; family support to participate in vaccine trials and desire for economic incentives were significantly associated with WTP. *Conclusions*. There was a high proportion of WTP in HIV vaccine trials among Chinese MSM. The high HIV prevalence and high proportion of risky sexual behavior indicate that Liaoning MSM are potential candidates for HIV vaccine trials.

## 1. Introduction

Over the past thirty years, the HIV epidemic has gained momentum in many parts of the world despite continuing prevention efforts. There are estimated 34 million people living with HIV (PLHIV) around the world [1] and 780,000 in China [2]. In China, transmission among men who have sex with men (MSM) has increased rapidly. In 2007, 12.2% of the estimated new HIV infections were through male homosexual contact [3], but this proportion rose to 29.4% in 2011 [2]. Previous prospective cohort surveys have revealed high levels of HIV incidence among Chinese MSM (8.09/100 PY [4] in Beijing city, 4.17/100 PY [5] in Nanjing city, 7.1/100 PY [6] in Shenyang city, and 6.67/100 PY [7] in Yangzhou city). Additionally China has an estimated 17.8 million sexually active high risk MSM [8], so urgent action is needed to control the HIV epidemic among this vulnerable population [9]. The best long-term hope to control the pandemic may lie in the availability of a safe and effective preventive HIV vaccine. A 2009 study found a vaccine protection efficacy of 31.2% for an HIV vaccine trial in Thailand [10]. There are currently two Chinese-developed vaccines being tested in phases I and II trials in China [11]. Understanding WTP in HIV vaccine trials among priority populations is important for establishing strategies for participant recruitment and retention. Traditional studies investigating attitudes and behaviors via face-to-face interview may be biased by social desirability bias [12]. To get more accurate and reliable information about the MSM's WTP in HIV vaccine clinical trials, the current study utilized computer assisted self-interviewing (CASI) to reduce social desirability bias [13]. This study was conducted in Liaoning province, where a previous cohort study revealed both high HIV seroincidence (5.4 per 100 person-years, 95% CI: 2.0–11.3%) [14] and high prevalence (8.7%) among local MSM participants [15].

## 2. Material and Methods

From July 2010 to February 2011, a cross-sectional study was conducted among MSM in three cities in northeast China (Shenyang, Fushun, and Anshan). Study participants were recruited using three methods: (1) people who came to the voluntary counseling and testing (VCT) clinic for HIV testing were encouraged to participate in the study; (2) three peer recruiters were hired and trained to recruit MSM to attend the survey; (3) recruited participants were encouraged to refer their peer friends to participate in this study. 

Participant eligibility criteria included being male, being 18 years of age or older, having anal or oral sex with another male in the past 12 months, and not self-reported as HIV positive.

After written informed consent was obtained from participants, trained interviewers gave a brief introduction about HIV vaccines, including a description of clinical trials, and provided information on current progress and development of international vaccine research and the importance of developing a preventive HIV vaccine. After the HIV pretest counseling was implemented, 5 mL venous blood was drawn from each participant. HIV posttest counseling was provided to each MSM individual when they returned for their HIV test results within one week of blood drawn. The study protocol and informed consent forms were approved by the Institutional Review Board of the first affiliated hospital of China Medical University.

### 2.1. Data Collection

Data were collected using CASI in a private room. Each study participant was assigned a unique personal identity number that was linked to the questionnaire and HIV test results. Data were collected based on sociodemographics (age, income, ethnicity, education, employment, and marital status) and past 3-month sexual behavior including with male and female and regular and casual partners. The questionnaire also included a list of statements about WTP in hypothetical HIV vaccine clinical trials and motivators or perceived benefits and barriers or perceived risks to participate in HIV vaccine trials. 

### 2.2. Laboratory Tests

Serum samples were screened for HIV antibodies by enzyme-linked immunosorbent assay (ELISA; Vironostika HIV-1 Microelisa System; BioMérieux, Durham, NC), and positive samples were confirmed by HIV-1/2 Western blot assay (HIV Blot 2.2 WB; Genelabs Diagnostics, Singapore).

### 2.3. Data Analysis

Data were analyzed using SPSS (SPSS version 16.0, IL, USA). Bivariate analyses including chi-square test (categorical variables) and Wilcoxon two-sample tests (nonnormal continuous variables) were performed to evaluate associations of WTP with demographic characteristics, sexual behaviors, and perceptions and concerns regarding participation at an early stage of an HIV preventive vaccine clinical trial. Variables significant with *P* < 0.1 were fitted in a stepwise multivariate logistic regression model to estimate factors associated with WTP, and only factors with *P* < 0.05 were kept in the final fitted multivariate model. Two-sided *P* value less than 0.05 was considered statistically significant in this survey. 

## 3. Results

### 3.1. Demographic Characteristics

There were 626 participants who met the eligibility criteria and completed the CASI. The median age was 27 years, with a range of 18–76 years; 87.4% were of Han ethnicity and 9.7% were Man ethnicity; 67.3% were single, 15.5% were married, 10.7% cohabited with a female sex partner, and 5.9% were divorced; 33.9% finished college or higher levels of education, 32.7% completed senior high school education, and 33.4% completed junior high school education; 23.0% were unemployed; and the mean monthly income was 1,486 RMB (about 240$USD).

### 3.2. HIV Infection and Behavioral Characteristics

There were 31 (5.0%) participants who tested HIV-1 antibody positive. In the past three months, 48.7% participants' male sexual partners were sought through Internet, 28.9% at parks or public toilets, 10.1% at bathhouses or public washrooms, and 6.5% at bars. Almost all (96.5%, *n* = 604) had insertive or receptive anal intercourse with a male during the past three months, 65.5% (*n* = 410) had insertive or receptive anal intercourse with regular male partners. Meanwhile, in the past three months less than half (49.3%) reported always using condoms with their sex partners. More than half (54.8%, *n* = 343) had insertive or receptive anal intercourse with occasional male partners and of these 52.2% (*n* = 179) always used condoms with their sex partners. Additionally, 17.7% (*n* = 111) had sex with female partners and of these 79.3% (*n* = 88) reported always using condoms in the past 3 months.

### 3.3. WTP and Associated Factors

Overall 76.7% (95% CI: 73.2–79.9) (*n* = 480) of participants were WTP in HIV vaccine trials. Bivariate analyses found the following factors had marginally statistical association with WTP (*P* < 0.1): age, having sex with a man in the last three months, occasionally using condom with occasional male partners, having ever heard of HIV vaccine trials, having thought that being a participant in an HIV vaccine trial is a contribution to society, and having thought that HIV vaccination is a means of protection for spouses and family, desire for economic incentives (Table 1).

As shown in Table 1, compared to those MSM participants who were non-WTP, those MSM participants who were WTP had a higher proportion of unprotected sex with both regular male sex partners (*P* = 0.012) and occasional male sex partners (*P* = 0.028), had a higher proportion of “self-perceived high risk of HIV infection” (*P* = 0.077), a higher proportion of “heard of HIV vaccine before the survey” (*P* = 0.002), a higher proportion of “thought being a participant in an HIV vaccine trial is a contribution to society” (*P* < 0.001), a higher proportion of “thought HIV vaccination is a means of protection for spouses and family” (*P* < 0.001), a higher proportion of “believed that their family (*P* < 0.001) and regular male partners” (*P* < 0.001) would support their HIV vaccine trial participation”, a higher proportion of “believed a vaccine could protect people from becoming infected with HIV” (*P* < 0.001), a higher proportion of “if known that the inoculation place is secret (*P* < 0.001) or a general hospital (*P* < 0.001)”, a lower proportion of “fear family member would discriminate them due to participation in a vaccine trial” (*P* < 0.001) and a lower proportion of “fear of sexual rejection due to participation in a vaccine trial” (*P* = 0.009) and “believed a vaccine may cause health problems” (*P* < 0.001).

### 3.4. Multivariate Logistic Regression Analysis of Factors Associated with WTP in HIV Preventive Vaccine Trials

Variables with *P* value less than 0.10 in univariate analysis were included in a stepwise multivariate logistic regression model. The variables which are significant at *P* < 0.05 in the multivariate logistic regression model were retained in the final model as shown in Table 2. The factors that were independently associated with WTP were the thought that HIV vaccination is a means of protection for spouses and family (aOR = 4.1, 95% CI: 1.7–9.9), the belief that their family would support their vaccine trial participation (aOR = 6.3, 95% CI: 2.3–17.2), and the desire for economic incentives (aOR = 7.3, 95% CI: 2.7–19.8).

## 4. Discussion

We found a high proportion (76.7%) of MSM were willing to participate in the hypothetical HIV vaccine trial. This proportion was higher than that found in studies in Thailand (58%), Australia (63%), Brazil (57%), and Beijing, China (71.9%) [16–19]. 

The main strengths of the study lie in the use of CASI to collect data from participants. To our knowledge this is the first study that has used CASI to examine WTP in a hypothetical HIV vaccine trial among MSM in China. It is believed that participants are more likely to report accurately with CASI compared with traditional face-to-face interviewing [13]. 

The prevalence of HIV in the current study was 5.0% which is close to prevalence found in Shenyang in 2006 (5.7%) [20] and 2008 (8.7%) [15], which implies that the HIV epidemic among MSM in Liaoning is still serious. The high proportion of WTP for HIV vaccine trials indicates that Liaoning MSM are promising candidates for HIV vaccine clinical trials.

Through univariate analysis we found that those who were WTP had a higher proportion of unprotected sex with regular (*P* = 0.012) or occasional (*P* = 0.028) male sex partners and a higher proportion of “self-perceived high risk of HIV infection” (*P* = 0.077). The results indicate that the high-risk MSM were more willing to participate in HIV vaccine trials than lower-risk people [17, 21–24]. It is more helpful to recruit MSM participants with relative higher HIV infection risk in the assessment of vaccine efficacy for HIV prevention. VCT is a good way to make MSM realize that they are at risk of being infected with HIV [25–27]. Therefore, VCT sites may be one candidate place for recruiting MSM participants with higher HIV exposure risk to attend HIV vaccine trail.

Those who were WTP had a higher proportion of “thought being a participant in an HIV vaccine trial is a contribution to society” (89.4% versus 67.1%). It means that MSM who have social responsibility are more willing to participate, which is consistent with Newman et al.'s research in North America [23]. Those who were WTP had a higher proportion of “heard of HIV vaccine before the survey” (*P* = 0.002) and “believed a vaccine could protect people from becoming infected with HIV” (*P* < 0.001). It indicates that people who know more knowledge about HIV vaccine and want to get protection from the vaccine are more willing to participate [28]. Those who believed their regular male partners would support their HIV vaccine trial participation were more willing to participate (*P* < 0.001) and “fear of sexual rejection due to participation in a vaccine trial” (*P* = 0.009) maybe a barrier. It hints that sex partner's opinion may influence the WTP [29]. Those who were non-WTP had a higher proportion of “believed a vaccine may cause health problems” (*P* = 0.002), which agrees with the finding from Newman et al. and Kiwanuka et al.; they also found that the side effect would impede MSM's WTP [30, 31]. The finding implies that safety testing of the vaccine could be the first step and medical treatment preparation is needed. The inoculation place is secret or general hospital would increase WTP (*P* < 0.001) and indicate that inoculation place needs to protect MSM's privacy and also needs a comprehensive health care condition. Several strongly correlated variables in the univariate model failed to enter the multivariate model. It might be caused by the relatively small sample size of this survey.

The current study found that believing that HIV vaccination is a means of protection for their spouses and family (aOR = 4.1, 95% CI: 1.7–9.9) was significantly associated with WTP. The role of family in traditional Chinese culture may be an important component for WTP in HIV vaccine trials. MSM who do not want to transmit HIV to their families are more willing to attend preventive vaccine trials and similar findings were found in two other surveys [19, 32]. So this group of MSM who are more caring about the health of their family members should be placed as one of the priority subpopulations for HIV vaccine trail candidate. Additionally, those MSM who had a belief that family would support their HIV vaccine trial participation have a significant higher WTP (aOR = 6.3, 95% CI: 2.3–17.2). It shows that family support to attend HIV vaccine trail is critical, so the subgroup of MSM who have good family support to attend HIV vaccine trail should also be placed as another priority candidate for HIV vaccine trail.

Desire for economic incentives (aOR = 7.3, 95% CI: 2.7–19.8) was also found significantly associated with WTP in this study. Economic motivations have been found to be significantly associated with WTP in vaccine trials in other populations [22, 23]. Providing adequate incentives to participants in clinical trials is recommended [33]. However, incentive levels should be set at an appropriate amount to avoid financial coercion.

This study was subject to several limitations. The sample size is relatively small and the study was conducted in 3 cities of Liaoning Province, so these results may not be generalizable to all MSM in Liaoning Province or China. This was a cross-sectional survey, so causal associations between the studied outcome and exposure factors cannot be well determined. About one-third of the participants had not heard of HIV vaccines before attending this survey, so the reported WTP may be relatively unreliable.

## 5. Conclusions

There was a high proportion of WTP in HIV vaccine trials among Liaoning MSM. The high HIV prevalence and high proportion of risky sexual behavior indicate that Liaoning MSM are potential candidates for HIV vaccine clinical trials. Research teams that are developing HIV vaccines should consider Chinese MSM as an important population to evaluate the protective effect of HIV vaccination.

## Figures and Tables

**Table 1:** Association between characteristics and willingness to participate (WTP) in HIV vaccine trials.

Variable	*n* = 480 WTP (%)	*n* = 146 Non-WTP (%)	*P* value
Median age (IQR)	26 (23–35)	28 (24–43)	0.002
Han ethnicity	86.3	91.1	0.123
Married or cohabited with female sex partners	31.3	37.7	0.148
Finished college or higher levels of education	33.8	34.3	0.624
Employed	22.3	25.3	0.443
Median monthly income (IQR, RMB)	1500 (1000–2000)	1200 (1000–2000)	0.771
Found male sex partners using the Internet	49.4	46.6	0.068
Had sex with a man in the last 3 months	95.6	99.3	0.034
Had more than 2 male sex partners in the last 3 months	38.8	37.0	0.701
Never used condoms with regular male sex partners	16.9	4.9	0.012
Used condoms occasionally with occasional male sex partners	21.6	15.9	0.028
Had sex with a female in the last 3 months	16.5	21.9	0.130
Self-perceived high risk of HIV infection	13.1	6.8	0.077
Heard of HIV vaccine before the survey	64.2	50.0	0.002
Thought being a participant in an HIV vaccine trial is a contribution to society	89.4	67.1	<0.001
Thought HIV vaccination is a means of protection for spouses and family	93.5	68.5	<0.001
Believed that their family would support their HIV vaccine trial participation	75.6	16.4	<0.001
Believed their regular male partners would support their HIV vaccine trial participation	74.4	15.8	<0.001
Fear family member would discriminate them due to participation in a vaccine trial	22.3	39.0	<0.001
Fear of sexual rejection due to participation in a vaccine trial	14.2	23.3	0.009
Believed a vaccine could protect people from becoming infected with HIV	71.9	47.9	<0.001
Believed a vaccine may cause health problems	4.6	11.6	0.002
If known that the inoculation place is secret	90.0	69.9	<0.001
If known that the inoculation place is general hospital	65.8	44.5	<0.001
Desire for economic incentives	71.0	14.1	<0.001

**Table 2:** Multivariate logistic regression analysis of factors associated with WTP in HIV preventive vaccine trials.

Independent factors	*N*	WTP (%)	aOR* (95% CI)	*P* value
Thought HIV vaccination is a means of protection for spouses and family				
No	77	31 (40.3)	4.1 (1.7–9.9)	0.002
Yes	549	449 (81.8)		
Desire for economic incentives				
No	264	139 (52.7)	6.3 (2.3–17.2)	<0.001
Yes	362	341 (94.2)		
Believed that their family would support their HIV vaccine trial participation				
No	239	117 (49.0)	7.3 (2.7–19.8)	<0.001
Yes	387	363 (93.8)		

*aOR: adjusted odds ratio.

## References

[1] UNAIDS and WHO. GLOBAL HIV/AIDS RESPONSE Epidemic update and health sector progress towards Universal Access Progress Report 2011. http://www.who.int/hiv/pub/progress_report2011/summary_en.pdf.

[2] Ministry of Health, People’s Republic of China (2012). 2011 estimates for the HIV/AIDS epidemic in China. *Chinese Journal of AIDS & STD*.

[3] Ministry of Health 2009 estimates for the HIV/AIDS epidemic in China: Ministry of Health, People’s Republic of China, Joint United Nations Programme on HIV/AIDS. http://www.unaids.org.cn/en/index/Document_view.asp?id=413.

[4] Li D, Li S, Liu Y (2012). HIV incidence among men who have sex with men in Beijing: a prospective cohort study. *BMJ Open*.

[5] Hao C, Yan H, Yang H (2011). The incidence of syphilis, HIV and HCV and associated factors in a cohort of men who have sex with men in Nanjing, China. *Sexually Transmitted Infections*.

[6] Han X, Xu J, Chu Z (2011). Screening acute hiv infections among chinese men who have sex with men from voluntary counseling & testing centers. *PLoS ONE*.

[7] Peng Z, Yang H, Norris J (2012). HIV incidence and predictors associated with retention in a cohort of men who have sex with men in Yangzhou, Jiangsu Province, China. *PLoS ONE*.

[8] Zhang B, Li X, Shi T, Yang L, Zhang J (2002). Estimates of China’s gay /bisexual population and prevalence of HIV. *Chinese Journal of STD & AIDS Prevention and Control*.

[9] Shang H, Xu J, Han X, Spero Li J, Arledge KC, Zhang L (2012). HIV prevention: bring safe sex to China. *Nature*.

[10] Rerks-Ngarm S, Pitisuttithum P, Nitayaphan S (2009). Vaccination with ALVAC and AIDSVAX to prevent HIV-1 infection in Thailand. *The New England Journal of Medicine*.

[11] Phase-II clinical trial of self-developed HIV vaccine was started in China. http://www.chinanews.com/jk/hyxw/news/2009/03-21/1611799.shtml.

[12] John LK, Loewenstein G, Prelec D (2012). Measuring the prevalence of questionable research practices with incentives for truth telling. *Psychological Science*.

[13] Slack WV, Kowaloff HB, Davis RB, Delbanco T, Locke SE, Bleich HL (2011). Test-eretest reliability in a computer-based medical history. *Journal of the American Medical Informatics Association*.

[14] Xu J-J, Zhang M, Brown K (2010). Syphilis and HIV seroconversion among a 12-month prospective cohort of men who have sex with men in Shenyang, China. *Sexually Transmitted Diseases*.

[15] Chu ZX, Ma N, Xu JJ (2011). HIV prevalence and its associated factors among 2074 men who have sex with men(MSM) in Liaoning province, China. *Chinese Journal of Public Health*.

[16] Vieira De Souza CT, Lowndes CM, Landman Szwarcwald C, Sutmöller F, Bastos FI (2003). Willingness to participate in HIV vaccine trials among a sample of men who have sex with men, with and without a history of commercial sex, Rio de Janeiro, Brazil. *AIDS Care*.

[17] Newman PA, Roungprakhon S, Tepjan S, Yim S (2010). Preventive HIV vaccine acceptability and behavioral risk compensation among high-risk men who have sex with men and transgenders in Thailand. *Vaccine*.

[18] Van de Ven P, Mao L, Crawford J (2005). Willingness to participate in HIV vaccine trials among HIV-negative gay men in Sydney, Australia. *International Journal of STD and AIDS*.

[19] Li Q, Luo F, Zhou Z (2010). Willingness to participate in HIV vaccine clinical trials among Chinese men who have sex with men. *Vaccine*.

[20] Wang HL, Zhang M, Hu QH (2008). Prevalence of HIV/STD and risk behavior among men who have sex with men in Shenyang. *Chinese Journal of Public Health*.

[21] Newman PA, Duan N, Rudy ET, Roberts KJ, Swendeman D (2004). Posttrial HIV vaccine adoption: concerns, motivators, and intentions among persons at risk for HIV. *Journal of Acquired Immune Deficiency Syndromes*.

[22] Newman PA, Duan N, Lee S-J (2006). HIV vaccine acceptability among communities at risk: the impact of vaccine characteristics. *Vaccine*.

[23] Newman PA, Lee S-J, Duan N (2009). Preventive hiv vaccine acceptability and behavioral risk compensation among a random sample of high-risk adults in los angeles (La voices): research briefs. *Health Services Research*.

[24] Lee S-J, Brooks RA, Newman PA, Seiden D, Sangthong R, Duan N (2008). HIV vaccine acceptability among immigrant Thai residents in Los Angeles: a mixed-method approach. *AIDS Care*.

[25] Zou H, Wu Z, Yu J (2013). Internet-facilitated, voluntary counseling and testing (VCT) clinic-based HIV testing among men who have sex with men in China. *PloS ONE*.

[26] Yuntadilok N, Timmuang R, Timsard S (2013). Eroding gains in safe sex behavior, HIV/AIDS knowledge, and risk perceptions among royal Thai Navy conscripts after 28 years of the
AIDS epidemic in Thailand. *AIDS and Behavior*.

[27] Zhang X, Wang C, Hengwei W (2007). Risk factors of HIV infection and prevalence of co-infections among men who have sex with men in Beijing, China. *AIDS*.

[28] Asiki G, Abaasa A, Ruzagira E (2013). Willingness to participate in HIV vaccine efficacy trials among high risk men and women from fishing communities along Lake Victoria in Uganda. *Vaccine*.

[29] Chakrapani V, Newman PA, Singhal N, Jerajani J, Shunmugam M (2012). Willingness to participate in HIV vaccine trials among men who have sex with men in Chennai and Mumbai, India: a social ecological approach. *PloS ONE*.

[30] Newman PA, Daley A, Halpenny R, Loutfy M (2008). Community heroes or “high-risk” pariahs? Reasons for declining to enroll in an HIV vaccine trial. *Vaccine*.

[31] Kiwanuka N, Ssetaala A, Mpendo J High HIV-1 prevalence, risk behaviours, and willingness to participate in HIV vaccine trials in fishing communities on Lake Victoria, Uganda. *Journal of the International AIDS Society*.

[32] Yin L, Zhang Y, Qian H-Z (2008). Willingness of Chinese injection drug users to participate in HIV vaccine trials. *Vaccine*.

[33] Abraham NS, Young JM, Solomon MJ (2006). A systematic review of reasons for nonentry of eligible patients into surgical randomized controlled trials. *Surgery*.

